# Caring for the health and well-being of our learners in medicine as critical actions toward high-quality care

**DOI:** 10.1186/s13584-022-00517-w

**Published:** 2022-02-08

**Authors:** Orit Karnieli-Miller

**Affiliations:** Department of Medical Education, Sackler Faculty of Medicine, Tel Aviv University, Tel Aviv, Israel

## Abstract

A recent paper has focused on residents' poor lifestyle habits and their potential negative impact on patients' lifestyles. This commentary argues that there are even greater reasons to be concerned about the health and well-being of residents and medical students and the resultant effects on patients throughout the careers of these learners. There is a “hidden curriculum”, i.e., customs, rituals and norms of behavior, in medical education and in the training at the healthcare setting, often contradicts the formal curriculum and include messages that neglect the basic needs of the learners as well as the patients. Due to the impact of these messages on the professional identity formation of learners, including a deterioration in their own wellbeing as well as impairment of their ability to empathize with and care for patients, we must align our formal and hidden curricula to show dignity and caring for learners, colleagues, and patients. To do this well, we need to change our approach. We need to add processes for learners to support them in dealing with the stresses of their education and training and allow them to build their families and commit fully to medicine as a vocation, not just a job. We also must add faculty development processes to help align the formal and hidden curricula and help faculty empower and constructively assist their learners to handle challenging situations, e.g., where they see a resident struggling with patient care and day-to-day workload, through empathic feedback. When our learners are treated with kindness and respect they will lead more fulfilling lives and be better able to provide the high-quality care and caring all patients deserve.

A recent important paper addresses the issue of healthy behavior among medical students versus residents versus fully-trained physicians [1]. In this paper, Wilf-Miron, Kagan, and Saban compared pre-clinical students with the other groups using a cross–sectional, self-reported questionnaires. The findings are worrisome. Half of the residents experienced high levels of stress and had poorer habits of eating, such as eating fewer vegetables, eating more processed food, and drinking more sweetened beverages. Residents also smoked more cigarettes. Forty-three percent slept less than 5 h a night. This has led to concern, both for the health of the residents and for that of their patients.

The clinical years of medical school and the years of internship and residency are critical times where the professional identity as a physician is formed. Residency is considered a particularly challenging time. It is a time of intense learning and responsibility. It is the time when the learner must grow and develop into a competent, empathetic, trustworthy physician.

The “hidden curriculum”, which includes customs, rituals, and norms of behaviors, in clinical settings, has a strong impact on the professional identity formation of medical students and residents [2–5]. The hidden curriculum teaches learners about actual professional conduct versus what has been told to them about conduct [2, 3]; including the conduct in interactions with each other and with patients. The hidden curriculum in residency includes various messages. Two very prominent ones coming from more senior clinicians have the following character: “We, in the past, worked hard, and so should you, the current resident;” and “This is just a phase that will pass—you, as a resident should just stick up with the toll of residency, and then as you grow older and more experienced, you will have other privileges.” These perceptions, whether expressed verbally or in non-verbal behaviors, permit more senior physicians to neglect residents' basic needs over a 4–6 year period, see them as unjustifiably whiny, and expect them to toughen up as part of their professional development.

But does this have to be the case? Does this really toughen up learners in a good way? Does it help them develop into the professionals we want them to become?

As Wilf-Miron and colleagues [1] point out, studies have shown that a physician who has unhealthy habits tends to have less effective dialogues with patients to motivate them to have a healthy lifestyle. Similarly, we must be concerned that physicians may have more difficulty being empathic and truly caring for others if they have been treated with little empathy and caring for their own needs while in training.

Questions arise, such as can you act with compassion for a mistreated patient in the emergency room when your rights are not preserved? Can you relate empathically to the lack of sleep by the patient or for the long wait when you had no time to sleep, eat, or go to the bathroom?

Some studies show that empathy declines in the clinical years of medical school [6]. This deterioration does not need to occur [7]. When it occurs, it is not because our students lost their values or did not learn formally how to discuss suffering or be empathic; various processes exist for preserving values and teaching these skills. Rather, it is because the students are exposed to a problematic culture with little time to reflect and choose differently. They have no power to change that culture. Instead, they assimilate into that culture. Senior clinical students identify more and more with their near-peer colleagues, their future-self, i.e., residents. They look at the residents, see how hard they work, and how they experience time pressures that lead them to rush the patient care with little caring.

We, like others around the world [8], spend much effort and time teaching our medical students the importance of empathy and how to provide empathic care in various situations [9, 10]. This has some positive impact [7, 9, 11]. But this impact vanishes throughout the years when they do not see it happening much in practice, when the hidden or informal curriculum observed in the day-to-day routines contrasts what they have learned in the formal curriculum. Furthermore, the learners lose their will and energy to invest themselves in caring when they feel they are not treated with empathy. In recent years I have received more and more e-mails from students saying: "Thank you for teaching us how to care for our patients; this is helpful. Will you teach us also how to care for ourselves in times of stress, burden, and lack of sleep?" When we try to answer this request through lectures and workshops about self-care, well-being, and resiliency, we get some furious reactions from students, saying: "don't put a bandage on the problematic system you are exposing us to." Problematic indeed.

So what should we do?

We need to change. We need to align our formal curriculum that emphasizes the dignity and caring for students, colleagues, and patients, with our hidden and informal curriculum. We need to add more processes of helping see the student, intern, and resident as a person throughout their development.

One process would be to develop support groups of, and for, learners at each level of clinical training to help them deal with their personal and professional dilemmas. There are examples of these groups; and they are meant to help the learners reflect on their experiences and learn different tools to manage challenging encounters and provide high-quality care [12]. These groups can be done with learners, and with experienced physicians, to support them as a team and to enhance their understanding of how their actions impact each other.

We can add more reflective writing on the hidden curriculum experiences, to help identify these occurrences and deliberate to enhance learning [9, 13, 14]. It can be done within the support groups. It can focus on learning both from negative and from positive experiences, and on identification of role models. The process of searching for positive experiences can be very powerful and help bring enthusiasm and hope [15]. Other processes can include simulated encounters in a safe space. These encounters can raise the challenges of the hidden curriculum, more openly discuss them, address ways to overcome them [16, 17], and advance moral courage in addressing these [18].

These approaches should be ongoing. They should be done in undergraduate, graduate, and continuing education to truly help learners identify their personal and professional developmental processes, manage their life stressors, build families, reflect on their experiences, and commit to their and the profession’s values. All of this should result in their having a strong commitment to a profession that is not merely work, nor simply a career, but a vocation that treats its people and, in turn, their patients, with kindness and respect.

We often tell our students, “you will be the change,” “'you learned things differently;” but we more and more understand that this is a responsibility they cannot take on by themselves. A longitudinal dissertation done by Neufeld Kroszynski, showed, sadly, that when time passes and after students have been exposed to the medical culture and to the hidden curriculum around them, they often become what they feared they would be: They find themselves acting with little compassion, or violating some patients' rights [19].

Therefore, to bring the culture change we also need to invest in faculty development. Focus on senior physicians, aligning the curriculums. Sharing with them the formal curriculum taught in the medical school and identify the gaps between it and the hidden curriculum that clinical learners experience. We can develop faculty by teaching them constructive ways to handle challenging situations, e.g., where they see a resident struggling with patient care and day-to-day workload; by helping them practice an empathic feedback process that includes focus on ways to help the learner manage these tasks; and by enhancing their own teamwork and caring skills. These can be accomplished through techniques such as observed structured/standardized teaching evaluation and debriefing discussions.

Wilf-Miron and colleagues suggested some curricular changes, including a recommendation that there be “a mandatory course to provide knowledge on lifestyle medicine to all medical students." [1] I want to emphasize that the issue is not just knowledge, nor acknowledgment of the danger of an unhealthy lifestyle, or telling learners about the need for empathy. The key issues are helping learners manage the various demands on them, encouraging them to speak up and share concerns about their, and their patients', health, needs, and rights; and helping our faculty become role models for caring. We, the educators, need to acknowledge these key issues if we are going to bring the long overdue change and help learners develop and become the professionals we aspire them to be.

In the Hebrew language, we refer to a professional with two words referring to person and to profession (“Anshei Mikzoa”). Thus, we acknowledge that professionals are first and foremost people. This should remind us that we need to invest in caring for the person, the professional. Treating learners with care and compassion will help them develop into professionals who can treat others wholeheartedly and care for others’ health and well-being. To do this, we must uncover and deal with the hidden curriculum. We must identify it, reflect on it, and help those who promulgate it to change so that it is aligned with our formal curriculum. We need to invest in relationships and care that will lead to improving the health and well-being of all healthcare professionals and, as a result, improve the healthcare of the patients they serve.
